# A snapshot of public knowledge of novel coronavirus disease 2019: a web-based national survey

**DOI:** 10.1186/s12889-021-10495-4

**Published:** 2021-03-09

**Authors:** Yu Liu, Dan Wang, Hao Xu, Ying Xiao, Cui Chen, Ru-Nan Chen, Liang-Hao Hu, Zhao-Shen Li

**Affiliations:** 1 Department of Gastroenterology and Hepatology, Jinling Hospital, Medical School of Nanjing University, Nanjing, Jiangsu China; 2 Department of Gastroenterology, Changhai Hospital, The Second Military Medical University, 168 Changhai Road, Shanghai, China; 3 Department of Infectious Diseases, Changhai Hospital, The Second Military Medical University, Shanghai, China; 4 Department of Nursing, Changhai Hospital, The Second Military Medical University, Shanghai, China; 5 Department of Nursing, The Second Military Medical University, Shanghai, China

**Keywords:** Coronavirus disease 2019, Transmission, Psychological state, Control, Prevention

## Abstract

**Background:**

Although the number of existing cases of coronavirus disease 2019 (COVID-19) in China has been decreasing since late February 2020, the number of confirmed cases abroad is surging. Improving public knowledge of COVID-19 is critical to controlling the pandemic. This study aimed to determine China’s public knowledge of COVID-19 and the attitudes towards control measures.

**Methods:**

A cross-sectional study was conducted over 48 h from 22:30 29 February 2020 to 22:30 2 March 2020 based on a self-administered web-based questionnaire. The survey was conducted on the WeChat network. Exponential non-discriminative snowball sampling was applied. The questionnaire was voluntarily completed by WeChat users. The questionnaire covered basic demographic information, public knowledge of the epidemiological and clinical characteristics of COVID-19, psychological state, and attitudes towards the overall control measures. The primary outcome was the China’s public knowledge of COVID-19 and the attitudes towards control measures and secondary outcome was the psychological state of the public during this pandemic.

**Results:**

The study included 10,905 participants and 10,399 valid questionnaires were included for analysis. Participants with tertiary education, younger participants and healthcare workers had better overall knowledge than other participants (all *P* < 0.05). Approximately 91.9% of the participants believed in person-to-person transmission and 39.1% believed in animal-to-person transmission. No significant correlation between anxiety and the number of regional existing confirmed cases was found, while participants in Hubei were more anxious than those in other regions. In general, 74.1% of the participants acknowledged the effectiveness of the overall control measures, and the percentage of participants with agreement with the overall control measures was negatively correlated with the number of regional existing confirmed cases (*r* = − 0.492, *P* = 0.007).

**Conclusions:**

In conclusion, the survey revealed that the Chinese public had overall good knowledge of COVID-19 except for those indeterminate knowledges. With dynamic changes in the global pandemic situation and more research, further studies should be conducted to explore changes in public knowledge and attitudes towards COVID-19 in the future. The media could be used in a strict and regular manner to publicize knowledge of such pandemics to halt their spread.

**Supplementary Information:**

The online version contains supplementary material available at 10.1186/s12889-021-10495-4.

## Introduction

On 31 January 2020, the World Health Organization (WHO) declared the coronavirus disease 2019 (COVID-19) to be a public health emergency of international concern and the risk assessment was very high at both the Chinese and global level [1]. According to the COVID-19 situation report of the WHO, as of 29 February 2020, there were 79,394 confirmed cases across China and 85,403 confirmed cases globally [2]. The number of existing cases in China began to decrease on 19 February 2020, however, the number of confirmed cases abroad has surged since late February.

The first case of this pneumonia was diagnosed on 8 December 2019 with an unknown pathogen in Wuhan, Hubei, China [3]. One month later, the novel coronavirus was identified as the pathogen leading to the pandemic. In the following days, a great amount of massive news appeared in the media. The epidemiological and clinical characteristics of COVID-19 were released by the Chinese Center for Disease Control and Prevention through various social media channels, such as web pages on cellphones and computers, Television news, and real-time radio broadcasts [4]. To draw more public attention, great efforts were made by the government. News about COVID-19 was broadcast all day and night. The front-line situation of the COVID-19 pandemic in Wuhan was even broadcast on the most important traditional Television shows—the Spring Festival Gala of China Central Television. These shows used the most serious language to arouse people’s attention and to urge the whole nation to unite together to win the battle against COVID-19. Grassroots cadres patrolled in the streets and towns to tell people to stay home and not gather outside.

To control the pandemic, the General office of the State Council announced the prolongation of the Spring Festival holiday [4]. All restaurants, shopping malls, cinemas, and entertainment venues were closed in many cities during the Spring Festival. Mass events were cancelled. Companies delayed the resumption of work and schools delayed the start of the spring semester. Numerous cities and towns were shut down. Body temperature screenings were requested at the entrances of train stations, bus stations and airports. Quarantine for at least 14 days was compulsory for people from other cities.

Improving public knowledge of the disease, its transmission patterns and effective protective measures is the foundation for the control and prevention of the disease. However, discrepancies may exist in the knowledge held by people of varying socioeconomic levels or ages. In addition, the pandemic may have negative effects on individuals’ psychological state. Therefore, investigating the public’s awareness of COVID-19 and its psychological state could help in identifying the public knowledge gap and potential psychological disorders so that more targeted efforts could be made to increase public awareness and decrease the risk of psychological disorders.

This study aimed to determine the China’s public knowledge of COVID-19 and psychological state as well as attitudes towards control measures via a quick online survey.

## Materials and methods

A cross-sectional study was conducted over 48 h from 22:30 29 February 2020 to 22:30 2 March 2020 based on a self-administered questionnaire. The questionnaire was created and released based on the Sojump (Changsha Ranxing Information Technology Co., Ltd., Changsha, China, http://www.sojump.com) online survey tool and distributed to participants via WeChat (Tencent Inc., Shenzhen, China, Version 7.0.12), a popular social media platform.

### Questionnaire design

The questionnaire was entitled “Public knowledge of the coronavirus disease 2019 (COVID-19)”. Based on a literature review, the questionnaire covered the following major aspects: basic demographic information (including gender, age, marital status, educational level, occupation, etc.), basic public knowledge of the epidemiological characteristics (the nature of the disease, transmission, symptoms, therapies, etc.), personal protection measures, psychological state, and attitudes towards control measures [3–5]. In total, 17 groups of questions with one or more correct answers were constructed.

To ensure the accuracy of the results, several mutually exclusive questions were established to test the validity of the questionnaire and contradictory answers were obtained to exclude invalid questionnaires. A pilot test of the questionnaire was performed among 20 participants with different ages and jobs in different provinces to establish the validity of the content of the questionnaire and to ensure its comprehensibility and feasibility. Based on the pilot test, the language of the questionnaire was adjusted to be concise and sufficiently unequivocal. The Cronbach alpha value for the questionnaire was 0.990, which was considered satisfactory for the survey. The final formal questionnaire that was released on Sojump (https://www.wjx.cn/jq/60631081.aspx) can be found in the supplementary material (Additional file 1).

### Samples and survey methods

WeChat is the social media platform that is the most widely and frequently used by Chinese people [6]. More than 1.15 billion customers are active users of WeChat, and they are distributed across more than 200 countries with 20 different languages [6, 7]. Thus, the survey was conducted on the WeChat network. Exponential non-discriminative snowball sampling was applied in this survey. The questionnaire was first released at 22:30 29 February 2020, and data were collected at 22:30 2 March 2020.

The questionnaire was anonymous and did not contain any identifiable personal information. Every participant fully had the right and the freedom to complete the questionnaire or forward the link.

### Definition

To simplify the analysis, the awareness score was defined as the accuracy of knowledge of COVID-19, which was calculated by adding the correct answers related to the nature of the disease and its transmission, symptoms and therapies based on current knowledge. The specific questions included Q9 (A1, A2, A4, A5, A9), Q10, Q11, Q12 (A1, A2, A6, A7, A8) and Q13. Each correct answer was assigned a score of one, for a total of 22 points.

### Statistical analysis

The data from valid questionnaires were analyzed with SPSS 23.0 (version 23.0, SPSS, Chicago, Illinois, USA). Categorical variables are presented as percentages (numbers, n), and the differences among categorical variables were compared using the chi-square test or Fisher’s exact test when appropriate. The within-group comparison was made through the least significant difference test. The correlation between extreme anxiety and the number of regional existing confirmed cases and the correlation between high agreement with the overall control measures and the number of regional existing confirmed cases were analyzed by the Pearson correlation test with GraphPad Prism 8.0.2 (version 8.0.2, GraphPad Software Inc., California, USA). All tests were two tailed, and *P* < 0.05 was considered statistically significant.

## Results

### Basic demographic characteristics of the participants

The study included 10,905 participants recruited within 48 h. After excluding 566 invalid questionnaires with missing data or bad data quality, 10,399 questionnaires were included for analysis. Among the valid questionnaires, 9653 (98.1%) were completed by domestic participants and 192 (1.9%) were from abroad. Most participants were from southern China. As Hubei was the center of the pandemic, the characteristics of the participants were compared between Hubei and other regions. The basic characteristics are presented in Table 1.

**Table 1:** Basic characteristics of the 10,339 participants

Characteristics	Total	Hubei	Other regions
Male	4768 (46.1%)	512 (40.5%)	4265 (46.9%)
Age, y			
18–44	7888 (76.3%)	949 (75.1%)	6939 (76.5%)
45–59	2081 (20.1%)	277 (21.9%)	1804 (19.9%)
60–74	370 (3.6%)	38 (3.0%)	332 (3.7%)
Married	7539 (72.9%)	975 (77.1%)	6564 (72.3%)
Tertiary education	8347 (80.7%)	1034 (81.8%)	7313 (80.6%)
Healthcare workers	2588 (25.0%)	182 (14.4%)	2406 (26.5%)
Heard of COVID-2019	9845 (95.2%)	1197 (94.7%)	8648 (95.3%)

A total of 9845 (95.2%) participants had heard of the disease and the remaining 494 (4.8%) participants had not. Subsequent analysis was performed with data from the 9845 participants. The majority of the participants received information about COVID-19 from the Internet, followed by TV (Additional file 2). Less than 10% of the participants knew about the disease from other sources, including word of mouth (from family members, neighbors or grassroots cadres), newspapers or magazines and hospitals.

### Public knowledge of COVID-19

#### Public knowledge of the epidemiological characteristics of COVID-19

Overall, public knowledge regarding the epidemiological characteristics of COVID-19 was similar between participants from Hubei and other places.

In terms of the pathogen, 8505 (86.4%) participants thought that COVID-19 was caused by a virus. More participants from Hubei believed in fecal-oral transmission. A total of 91.9% (9046/9845) and 39.1% (3845/9845) of the participants believed that there was person-to-person transmission and animal-to-person transmission, respectively. In total, 58.2% (5727/9845) and 50.3% (4950/9845) of the participants believed that the virus could be transmitted through talking and shaking hands with others, respectively. Seniors were considered a susceptible population by 98.8% (9729/9845) of the participants. Only 65.7% (6465/9845) of the participants recognized children as susceptible population (Table 2).

**Table 2:** Knowledge of the epidemiological characteristics of COVID-19 of 9845 participants

Items	Answers	Total	Hubei	Other regions	P
Pathogens					0.546
Virus	√	8505 (86.4%)	1042 (87.1%)	7463 (86.3%)	
Bacteria	×	143 (1.5%)	12 (1.0%)	131 (1.5%)	
Both virus and bacteria	×	876 (8.9%)	103 (8.6%)	773 (8.9%)	
No idea	×	321 (3.3%)	40 (3.3%)	281 (3.2%)	
Transmission					
Droplet transmission	√	9647 (98.0%)	1176 (98.2%)	8471 (98.0%)	0.583
Aerosol transmission	√	7389 (75.1%)	907 (75.8%)	6482 (75.0%)	0.539
Fecal-oral transmission	○	6771 (68.8%)	856 (71.5%)	5915 (68.4%)	0.029
Contact transmission	√	9251 (94.0%)	1143 (95.5%)	8108 (93.8%)	0.018
Person-to-person transmission	√	9046 (91.9%)	1106 (92.4%)	7940 (91.8%)	0.488
Animal-to-person transmission	○	3845 (39.1%)	430 (35.9%)	3415 (39.5%)	0.018
Transmitted through talking with others	△	5727 (58.2%)	723 (60.4%)	5004 (57.9%)	0.095
Transmitted through shaking hands with others	△	4950 (50.3%)	625 (52.2%)	4325 (50.0%)	0.153
Susceptible population					
Children	√	6465 (65.7%)	693 (57.9%)	5772 (66.7%)	< 0.001
Young adults	√	4567 (46.4%)	532 (44.4%)	4035 (46.7%)	0.150
Seniors	√	9729 (98.8%)	1192 (99.6%)	8537 (98.7%)	0.006
Others viewpoints					
It’s a noncommunicable disease	×	4 (0.0%)	1 (0.1%)	3 (0.0%)	0.405
Infected persons could be asymptomatic	√	7879 (80.0%)	989 (82.6%)	6890 (79.7%)	0.017

The questions could be classified into the following three types based on the accuracy of the answers: (1) questions with a verified answer based on the current guidelines of COVID-19 and the literature. This type of answer was a definite true or false for one certain question, marked with “√” or “×”, respectively; (2) questions with a pending answer without solid proof as of the date of the current survey, marked with “○”; and (3) subjective questions with no unified right answers, representing only personal views, marked with “△”

#### Public knowledge of COVID-19 symptoms, therapies and personal protection measures

More than 95% of the participants recognized fever and cough as symptoms of COVID-19, and there was good consistency between participants from Hubei and other regions. Compared to participants from other regions, more participants from Hubei recognized debilitation as a symptom of COVID-19 (93.7% vs 91.3%, *P* = 0.004, Table 3). Fewer people in Hubei considered nasal congestion, rhinorrhea and sore throat to be symptoms of COVID-19 (all *P* < 0.01, Table 3).

**Table 3:** Knowledge of COVID-19 symptoms, therapies and prevention measures of 9845 participants

Items	Answers	Total	Hubei	Other regions	P
Recognition of symptoms					
Fever	√	9801 (99.6%)	1193 (99.7%)	8608 (99.5%)	0.533
Cough	√	9559 (97.1%)	1160 (96.9%)	8399 (97.1%)	0.683
Debilitation	√	9016 (91.6%)	1122 (93.7%)	7894 (91.3%)	0.004
Nasal congestion	√	4056 (41.2%)	413 (34.5%)	3643 (42.1%)	< 0.001
Rhinorrhea	√	4106 (41.7%)	406 (33.9%)	3700 (42.8%)	< 0.001
Sore throat	√	5673 (57.6%)	648 (54.1%)	5025 (58.1%)	0.009
Pantalgia	√	5325 (54.1%)	661 (55.2%)	4664 (53.9%)	0.401
Recognition of therapies					
There is no specific treatment and only symptomatic and supportive treatments help	√	8385 (85.2%)	1026 (85.7%)	7359 (85.1%)	0.572
There is specific drug to treat the disease	×	162 (1.6%)	20 (1.7%)	142 (1.6%)	0.904
Traditional Chinese medicine has a good therapeutic effect	○	1683 (17.1%)	229 (19.1%)	1454 (16.8%)	0.046
Integrative Chinese and western medicine is very effective	○	4581 (46.5%)	573 (47.9%)	4008 (46.3%)	0.322
Plasma antibodies in convalescent patients are effective	○	6184 (62.8%)	814 (68.0%)	5370 (62.1%)	< 0.001
The vaccine against COVID-19 is in use	×	566 (5.7%)	51 (4.3%)	515 (6.0%)	0.018
Traditional Chinese medicine (such as Shuanghuanglian) can prevent COVID-19	×	410 (4.2%)	52 (4.3%)	358 (4.1%)	0.740
No measures can prevent COVID-19	×	1103 (11.2%)	149 (12.4%)	954 (11.0%)	0.145
Personal prevention measures					
Wearing masks		9800 (99.5%)	1197 (100.0%)	8603 (99.5%)	0.005
Wearing goggles		2588 (26.3%)	396 (33.1%)	2192 (25.3%)	< 0.001
Frequent hand-washing		9522 (96.7%)	1163 (97.2%)	8359 (96.7%)	0.361
Daily home disinfection		4785 (48.6%)	644 (53.8%)	4141 (47.9%)	< 0.001
Covering mouth and nose when sneezing		6444 (65.5%)	771 (64.4%)	5673 (65.6%)	0.418
Measuring body temperature regularly		5645 (57.3%)	823 (68.8%)	4822 (55.8%)	< 0.001
No protection		6 (0.1%)	0 (0.0%)	6 (0.1%)	1.000

The questions could be classified into the following three types based on the accuracy of answers: (1) questions with a verified answer based on the current guidelines of COVID-19 and the literature. This type of answer was a definite true or false for one certain question, marked with “√” or “×”, respectively; (2) questions with a pending answer without solid proof as of the date of the current survey, marked with “○”; and (3) subjective questions with no unified right answers, representing only personal views, marked with “△”

Regarding therapies, most participants believed that there were no specific treatments for COVID-19 (85.2%), and only 5.7% (566/9845) considered there to be a specific vaccine to prevent the disease. A total of 46.5% (4581/9845) of the participants agreed with the efficacy of integrative Chinese and Western medicine. It seemed that compared to participants from other regions, more participants from Hubei had confidence in the efficacy of traditional Chinese medicine (19.1% vs 16.8%, *P* = 0.046). More participants from Hubei considered the plasma of convalescent patients to be an effective therapy (68.0% vs 62.1%, *P* < 0.001).

For 9845 participants, the overall awareness score was, on average, 17.8 ± 2.7. Young adults, participants who had received tertiary education, and healthcare workers had higher awareness scores than other participants (all *P* < 0.01, Table 4). In terms of regional differences, those from Hubei had lower overall awareness scores than those from other regions (16.9 ± 2.5 vs 17.1 ± 2.5, *P* = 0.01, Table 4). There was no difference in awareness score between those who were extremely anxious about the pandemic and those who were not or between those who highly agreed with the effectiveness of the overall control measures and those who did not (all *P* > 0.05).

**Table 4:** Awareness scores of 9845 participants

Items	n	Awareness score	P
Gender			< 0.001
Male	4540	17.620 ± 2.691	
Female	5305	17.980 ± 2.632	
Age			< 0.001
18–44	7616	17.892 ± 2.628	Ref.
45–59	1923	17.630 ± 2.752	< 0.001
60–74	306	17.026 ± 2.846	< 0.001
Marital status			< 0.001
Married	7135	17.883 ± 2.637	
Unmarried	2710	17.634 ± 2.730	
Occupation			< 0.001
Healthcare workers	2512	18.463 ± 2.467	
Others	7333	17.592 ± 2.694	
Education level			< 0.001
Tertiary	8089	18.049 ± 2.538	
Others	1756	16.731 ± 2.954	
Region			0.019
Hubei	1197	17.645 ± 2.602	
Others	8648	17.837 ± 2.673	
Source of first information			< 0.001
Internet	8112	17.867 ± 2.639	Ref.
Television	778	17.195 ± 2.757	< 0.001
Word of mouth^a^	723	17.736 ± 2.666	0.205
Others	232	18.297 ± 2.950	0.015
Concern much about the pandemic			< 0.001
Yes	8034	17.918 ± 2.618	
No	1811	17.353 ± 2.822	
Extremely anxious about the pandemic			0.341
Yes	1949	17.761 ± 2.776	
No	7896	17.827 ± 2.637	
Highly agreement with the effectiveness of control measures			0.735
Yes	7295	17.809 ± 2.657	
No	2550	17.829 ± 2.690	

^a^Word of mouth refers to: hearing information from family members, neighbors or grassroots cadres

### Psychological state regarding COVID-19

**Fig. 1:**
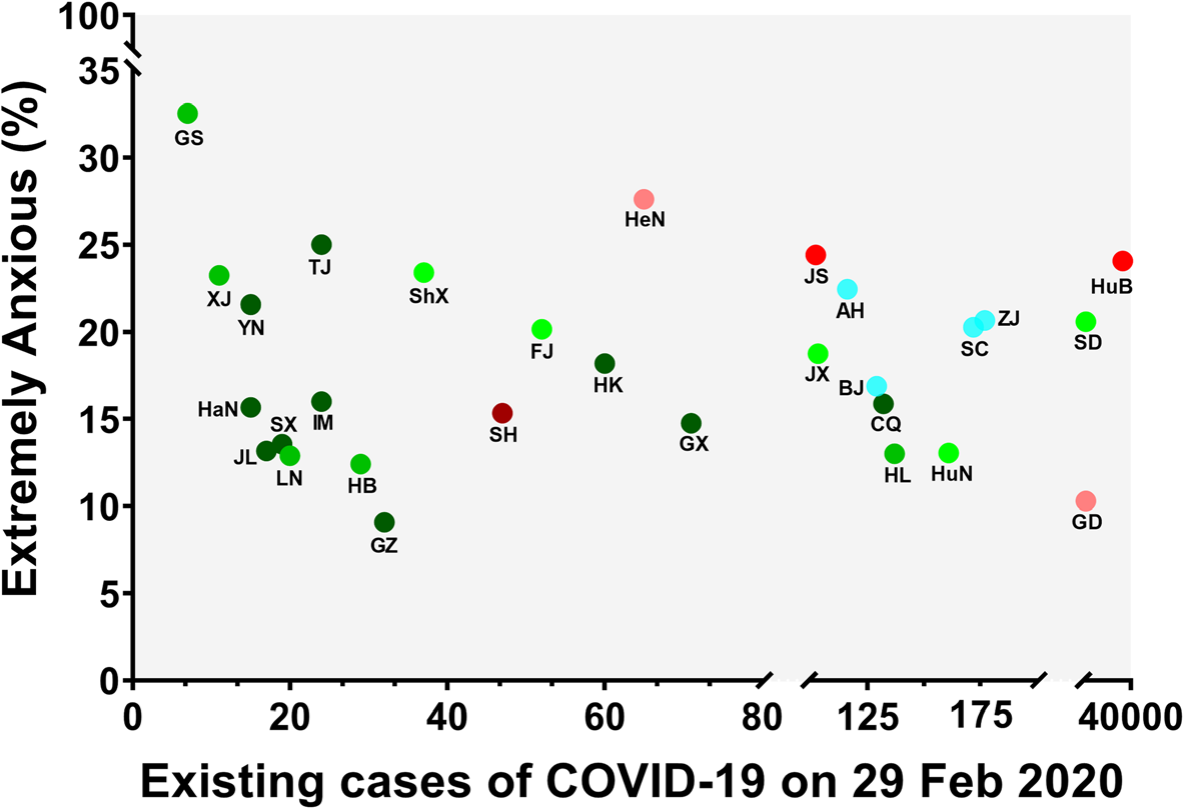
The correlation test. The correlation between extreme anxiety and the number of regional existing confirmed cases on 29 February 2020 (*r* = 0.178, *P* = 0.356). To reduce participant bias, regions from foreign counties and districts with no more than 10 participants were excluded from the correlation analysis

Approximately 28.2% (2777/9845, Table 5) of the population was extremely worried about being infected. More people worried about their relatives and friends than themselves (41.4% vs 28.2%, *P* < 0.001). Compared to participants in other regions, more participants in Hubei worried about themselves, relatives and friends being infected. Participants in Hubei were statistically more anxious and affected than those in other regions. However, there was no significant correlation between anxiety and the number of regional existing confirmed cases (Fig. 1, *r* = 0.193, *P* = 0.316).

**Table 5:** Psychological state regarding the pandemic and attitudes towards the systemic control measures for COVID-19 of 9845 participants

Items	Total	Hubei	Other regions	P
Psychological state regarding the pandemic				
Worried about self being infected				< 0.001
Extremely	2777 (28.2%)	402 (33.6%)	2375 (27.5%)	
Slightly	5783 (58.7%)	679 (56.7%)	5104 (59.0%)	
Never	1285 (13.1%)	116 (9.7%)	1169 (13.5%)	
Worried about relatives and friends being infected				< 0.001
Extremely	4075 (41.4%)	595 (49.7%)	3480 (40.2%)	
Slightly	5145 (52.3%)	560 (46.8%)	4585 (53.0%)	
Never	625 (6.3%)	42 (3.5%)	583 (6.7%)	
Being anxious about the pandemic				< 0.001
Extremely	1949 (19.8%)	288 (24.1%)	1661 (19.2%)	
Slightly	6345 (64.4%)	769 (64.2%)	5576 (64.5%)	
Never	1551 (15.8%)	140 (11.7%)	1411 (16.3%)	
Daily mood affected by the pandemic				< 0.001
Extremely	1897 (19.3%)	309 (25.8%)	1588 (18.4%)	
Slightly	6648 (67.5%)	768 (64.2%)	5880 (68.0%)	
Never	1300 (13.2%)	120 (10.0%)	1180 (13.6%)	
Paying close attention to news related to the pandemic				0.171
Extremely	8034 (81.6%)	988 (82.5%)	7046 (81.5%)	
Slightly	1771 (18.0%)	201 (16.8%)	1570 (18.1%)	
Never	40 (0.4%)	8 (0.7%)	32 (0.4%)	
Attitudes towards the control and prevention measures				
Staying at home during the Spring Festival is necessary				0.562
Highly agreement	9229 (93.7%)	1117 (93.3%)	8112 (93.8%)	
Agreement	582 (5.9%)	74 (6.2%)	508 (5.9%)	
Disagreement	34 (0.3%)	6 (0.5%)	28 (0.3%)	
Staying at home during the Spring Festival is effective				< 0.001
Highly agreement	8682 (88.2%)	965 (80.6%)	7717 (89.2%)	
Agreement	1071 (10.9%)	204 (17.0%)	867 (10.0%)	
Disagreement	92 (0.9%)	28 (2.3%)	64 (0.7%)	
Closing down shopping malls and cancelling mass events are necessary				0.002
Highly agreement	9067 (92.1%)	1127 (94.2%)	7940 (91.8%)	
Agreement	708 (7.2%)	58 (4.8%)	650 (7.5%)	
Disagreement	70 (0.7%)	12 (1.0%)	58 (0.7%)	
Closing down shopping malls and cancelling mass events are effective				0.084
Highly agreement	8724 (88.6%)	1077 (90.0%)	7647 (88.4%)	
Agreement	1063 (10.8%)	110 (9.2%)	953 (11.0%)	
Disagreement	58 (0.6%)	10 (0.8%)	48 (0.6%)	
The overall control measures are effective				< 0.001
Highly agreement	7295 (74.1%)	708 (59.1%)	6587 (76.2%)	
Agreement	2418 (24.6%)	444 (37.1%)	1974 (22.8%)	
Disagreement	132 (1.3%)	45 (3.8%)	87 (1.0%)

### Public attitudes towards the systematic control measures for COVID-19

**Fig. 2:**
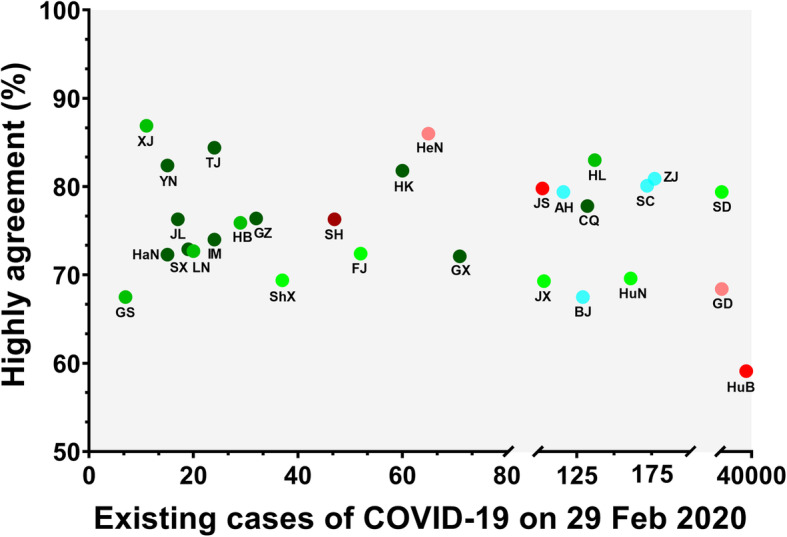
The correlation test. The correlation between highly agreement with the overall control measures and the number of regional existing confirmed cases on 29 February 2020 (*r* = − 0.492, *P* = 0.007). To reduce participant bias, regions from foreign counties and districts with no more than 10 participants were excluded from the correlation analysis

Staying at home during the Spring Festival was considered necessary by 93.7% of the participants, while 88.2% of the participants agreed with the effectiveness of this measure. Closing down shopping malls and cancelling mass events were considered necessary by 92.1% of the participants and 88.6% believed that these measures were effective. Overall, 74.1% (7295/9845) of the participants acknowledged that the overall control measures for this epidemic were effective, and only 1.3% did not. However, fewer people in Hubei acknowledged their effectiveness (59.1% vs 76.2%, *P* < 0.001). A moderate negative correlation was noticed between agreement with the overall control measures and the number of regional existing confirmed cases (Fig. 2, *r* = − 0.492, *P* = 0.007).

## Discussion

This study investigated the current Chinese public knowledge of COVID-19 via a 48-h Web survey. More than ten thousand people in China and elsewhere participated. Most of the participants had overall good knowledge of the disease, yet a knowledge gap was noticed in people of varying ages and educational levels. Residents of Hubei were more anxious than those of other regions. Highly agreement with the effectiveness of overall control measures was negatively correlated with the number of regional existing confirmed cases.

The overall knowledge of COVID-19 was satisfactory, which can be attributed to the broadcasting of the latest situation and scientific research. The media played a pivotal role in raising public awareness and improving people’s knowledge of COVID-19. According to our data, 82.4% of the participants learned about COVID-19 from the Internet. With the wide use of smartphones, the information that people obtained was abundant and updated in a timely manner [8, 9]. For example, people could gain access to real-time data on the number of confirmed cases reported by authorities and know what happened to a person in Wuhan, Hubei as soon as this information was posted on the web. With social media, the spread of information was explosive, in both speed and scale. Chinese Internet users spend more than 5 h online each day [10]. There is no doubt that people could gain more knowledge about the controlling and preventing of COVID-19. However, some information on social media was unverified and even consisted of rumors, which may mislead receivers and cause panic. Seniors and people without tertiary education may have more difficulty accepting scientific information and identifying rumors, contributing to the discrepancies in the knowledge obtained. The awareness of Hubei participants was not as good as that of participants from other regions, and the lower percentage of healthcare workers among Hubei participants could be the reason. In addition, compared to participants from other regions, the awareness of Hubei residents more often originated from personal experience, leading to subjective opinions about the disease.

It should be noted that more than half of the participants that there was fecal-oral transmission and agreed with the efficacy of plasma antibodies, which have not been verified with solid proof [3, 11–14]. The overreporting of related studies and specific cases made people too concerned about being infected. More than half of the participants believed that COVID-19 could be transmitted through talking with others or shaking hands with others. The overwhelming information on social media, the inability to distinguish between good information and bad information and the exaggerated reporting of specific cases contributed to these cognitive biases. These results suggest that the accuracy of media information during the spread of information should be improved.

Residents of Hubei were more anxious than those of other regions; however, the degree of anxiety had no correlation with the number of existing cases. The reasons for this result are as follows. First, the prevalence of COVID-19 in Hubei (32,959/59.17 million) was nearly 70 times that in the Hong Kong Special Administrative Region (60/7.45 million), a municipality with the second highest prevalence on 29 February, 2020. It was very likely that a resident in Hubei had relatives or acquaintances with COVID-19, which would greatly increase anxiety. For residents of other regions, infected people may just appear in the news. Second, the enormous medical burden in Hubei raised concerns about not being diagnosed and treated in a timely manner when people were sick. Third, people in Hubei were at the forefront of the pandemic and were the focus of people all around the world. The overwhelming online news and rumors about Hubei and even regional discrimination in China and elsewhere led to great anxiety.

The number of participants with highly agreement with the effectiveness of the overall control measures was moderately negatively correlated with the number of regional existing confirmed cases. Approximately 90% of the participants considered social distancing policies necessary and effective. However, there was a significant difference in highly agreement with the effectiveness of the overall control measures between the participants from Hubei and those from other regions. This result may be attributed to the extreme shortage of medical resources in Hubei when the outbreak occurred. In addition, the huge number of confirmed and suspected cases in Hubei upset residents, particularly the reports that the quarantine and control measures may have been initiated too late [15, 16]. According to a study on the prediction of the pandemic trend after public health interventions in mainland China, if control measures were implemented 5 days later, the scale of the outbreak might have tripled and if they were implemented 5 days earlier, the scale of the pandemic may have decreased to one-third [17].

This study has some limitations. The questionnaire was first released by the authors in a WeChat friends circle and then, the link was voluntarily forwarded by the authors’ friends. Thus, most participants were from Shanghai, where the study group was located, followed by cities around Shanghai in southern China. As a result, the percentages of healthcare workers and participants with tertiary education were much higher than the average level in China. In addition, some special questions related to the pandemic were not included in the questionnaire to avoid negative effects on the participants.

The current survey was initiated at the end of February and finished in 48 h. With the dynamic changes and information updatation in the pandemic situation in China and abroad, knowledge of and attitudes towards COVID-19 may change [18–21]. Moreover, our knowledge of the disease will improve as more researches are conducted. Further studies should be conducted to explore changes in public knowledge and attitudes in the future. The media have a great room to publicize knowledge of infectious diseases such as COVID-19 and prevention and control measures in the future to promote physical and psychological health, halt the spread of infectious diseases and conduct epidemiological studies. Nevertheless, the overwhelming information on social media, the inability to distinguish between good information and bad information and the exaggerated reporting of specific cases could contribute to cognitive bias and psychological disorders among members of the public. Thus, the accuracy of media information during the spread of the information should be improved.

## Conclusions

In conclusion, the survey revealed that the Chinese public had overall good knowledge of COVID-19 except for those indeterminate knowledges, which still needs to be elucidated. With dynamic changes in the global pandemic situation and more researches, further studies are needed to explore changes in public knowledge of and attitudes towards COVID-19 in the future. The media can be used in a strict and regular manner to improve people’s knowledge of epidemics, promote physical and psychological health, halt the spread of future epidemics and conduct epidemiological studies.

## Supplementary Information

**Additional file 1.** Questionnaire: Public knowledge of the coronavirus disease (COVID-19).

**Additional file 2.** Source of information regarding COVID-19 of 9845 participants.

## Data Availability

The datasets analyzed during the current study are available from the corresponding author upon reasonable request.

## Abbreviations

COVID-19: Coronavirus Disease 2019
WHO: World Health Organization
