# Supplementary material for: A snapshot of public knowledge of novel coronavirus disease 2019: a web-based national survey
Source: BMC Public Health. 2021 Mar 9;21:471. doi: 10.1186/s12889-021-10495-4 (PMC7940868; doi:10.1186/s12889-021-10495-4)
Supplement: Supplementary file 1 — Additional file 1. Questionnaire: Public knowledge of the coronavirus disease (COVID-19). [file 12889_2021_10495_MOESM1_ESM.docx]

**Additional file 1. Questionnaire:** **Public knowledge of the coronavirus disease (COVID-19).**

(This is a questionnaire about your knowledge and attitude about COVID-19. The questionnaire does not contain questions about your personal information. If you agree that the data could be collected, analyzed and published anonymously for non-commercial purposes, please fill in the questionnaire, otherwise please do not fill in the questionnaire.)

| **Choice (○, single choice; □, multiple choice)** | | | | |
| --- | --- | --- | --- | --- |
| Q1. What is your gender? | | | | |
| ○Male ○Female | | | | |
| Q2. How old are you? | | | | |
| ○18-44 ○45-59 ○60-74 | | | | |
| Q3. What is your education level? | | | | |
| ○ Elementary education, secondary education or none ○Tertiary education, including university education and college education | | | | |
| Q4. What category does your job belong to? | | | | |
| ○Health care workers ○Non- health care workers | | | | |
| Q5. What is your marital status? | | | | |
| ○Married ○Unmarried | | | | |
| Q6. Is there any health care worker in your family? | | | | |
| ○Yes ○No | | | | |
| Q7. Have you ever heard about the COVID-19 epidemic? | | | | |
| ○Yes ○No | | | | |
| Q8. The source you first heard about the COVID-19? |  | | | |
| ○News on cellphone ○News or programs on TV ○Family members ○Neighbors  ○Workers and cadres of the community or county office ○Newspaper ○During visit to hospital | | | | |
| Q9. In your point of view, the COVID-19 can be transmitted through the following ways: | | | | |
| □Cough droplets □Close contact □Oral-faecal route □Aerosol □Person to person  □Animal to person □Talking with others □Shaking hands with others □It cannot be transmitted | | | | |
| Q10. Whom do you think is vulnerable to COVID-19? |  | | | |
| □Children □Seniors □Young adults | | | | |
| Q11. Which of the following is correct in your point of view? |  | | | |
| □COVID-19 is caused by virus □COVID-19 is caused by bacteria □ Infected people could be asymptomatic | | | | |
| Q12. Which of the following about treatment and prevention of COVID-19 is correct in your point of view? | | | | |
| □There is no specific treatment and only symptomatic and supportive treatments help □ There is specific drug to treat the diseas | | | | |
| □Traditional Chinese medicine has a good therapeutic effect □Integrative Chinese and western medicine is very effective | | | | |
| □Plasma antibody in convalescent patients is effective □The vaccine against COVID-19 is in use | | | | |
| □Traditional Chinese medicine (such as Shuanghuanglian) can prevent COVID-19 □No measures could prevent COVID-19 | | | | |
| Q13. What are the signs and symptoms of COVID-19? |  | | | |
| □Fever □Cough □Weak □Nasal congestion □Rhinorrhea □Sore throat □Pantalgia □All of the above | | | | |
| Q14. Do you wear mask when you go out? | | | | |
| ○Yes ○No | | | | |
| Q15. What are the reasons that you don’t wear mask when going out? | | | | |
| □Lack of mask. □I am confident in not being infected. □It’s useless to wear mask. | | | | |
| Q16. What measures or protection are you taking to prevent infection? | | | | |
| □Wearing masks □Wearing goggles □Frequent hand-washing □Daily home disinfection  □Covering mouth and nose when sneezing □Measuring body temperature regularly □No protection | | | | |
| Q17. Please choose your opinion about the following viewpoint: | | | | |
| Viewpoints | | Highly Agreement | Agreement | Disagreement |
| I am worried about being infected. | | ○ | ○ | ○ |
| I am worried that my relatives and friends get infected. | | ○ | ○ | ○ |
| I am anxious about the epidemic. | | ○ | ○ | ○ |
| I am quite affected by the epidemic. | | ○ | ○ | ○ |
| I pay close attention to news of the epidemic. | | ○ | ○ | ○ |
| I think staying at home during Spring Festival is necessary. | | ○ | ○ | ○ |
| I think staying at home during Spring Festival is effective. | | ○ | ○ | ○ |
| I think it is necessary to close down shopping malls and cancel mass events. | | ○ | ○ | ○ |
| I think closing down shopping malls and cancelling mass events are effective. | | ○ | ○ | ○ |
| I think the overall control and prevention measures are effective. | | ○ | ○ | ○ |
