# Supplementary material for: A snapshot of public knowledge of novel coronavirus disease 2019: a web-based national survey
Source: BMC Public Health. 2021 Mar 9;21:471. doi: 10.1186/s12889-021-10495-4 (PMC7940868; doi:10.1186/s12889-021-10495-4)
Supplement: Supplementary file 2 — Additional file 2. Source of information regarding COVID-19 of 9845 participants. [file 12889_2021_10495_MOESM2_ESM.docx]

**Additional file 2. Source of information regarding COVID-19 of 9,845 participants**

|  | **Source of information** | | | | | |
| --- | --- | --- | --- | --- | --- | --- |
| **Items** | **Internet** | **Television** | **Word of mouth*** | **Newspaper or magazine** | **Hospital** | **P** |
|  | 8,112 (82.4%) | 778 (7.9%) | 723 (7.3%) | 36 (0.4%) | 196 (2.0%) |  |
| Gender |  |  |  |  |  | <0.001 |
| Male | 3,753 (82.7%) | 406 (8.9%) | 284 (6.3%) | 13 (0.3%) | 84 (1.9%) |  |
| Female | 4,359 (82.2%) | 372 (7.0%) | 439 (8.3%) | 23 (0.4%) | 112 (2.1%) |  |
| Age |  |  |  |  |  | <0.001 |
| 18-44 | 6,482 (85.1%) | 417 (5.5%) | 537 (7.1%) | 19 (0.2%) | 161 (2.1%) |  |
| 45-59 | 1,431 (74.4%) | 277 (14.4%) | 164 (8.5%) | 16 (0.8%) | 35 (1.8%) |  |
| 60-74 | 199 (65.0%) | 84 (27.5%) | 22 (7.2%) | 1 (0.3%) | 0 (0.0%) |  |
| Occupation |  |  |  |  |  | <0.001 |
| Healthcare workers | 2,034 (81.0%) | 164 (6.5%) | 139 (5.5%) | 11 (0.4%) | 164 (6.5%) |  |
| Others | 6,078 (82.9%) | 614 (8.4%) | 584 (8.0%) | 25 (0.3%) | 32 (0.4%) |  |
| Marital status |  |  |  |  |  | <0.001 |
| Married | 5,803 (81.3%) | 620 (8.7%) | 542 (7.6%) | 25 (0.4%) | 145 (2.0%) |  |
| Unmarried | 2,309 (85.2%) | 158 (5.8%) | 181 (6.7%) | 11 (0.4%) | 51 (1.9%) |  |
| Education |  |  |  |  |  | <0.001 |
| Tertiary | 6,735 (83.3%) | 542 (6.7%) | 596 (7.4%) | 29 (0.4%) | 187 (2.3%) |  |
| Others | 1,377 (78.4%) | 236 (13.4%) | 127 (7.2%) | 7 (0.4%) | 9 (0.5%) |  |
| Region |  |  |  |  |  | <0.001 |
| Hubei | 873 (72.9%) | 74 (6.2%) | 185 (15.5%) | 10 (0.8%) | 55 (4.6%) |  |
| Others | 7,239 (83.7%) | 704 (8.1%) | 538 (6.2%) | 26 (0.3%) | 141 (1.6%) |  |

* Word of mouth refers to: hearing information from family members, neighbors or grassroots cadres.
